# *FTO *gene variation and measures of body mass in an African population

**DOI:** 10.1186/1471-2350-10-21

**Published:** 2009-03-05

**Authors:** Branwen J Hennig, Anthony J Fulford, Giorgio Sirugo, Pura Rayco-Solon, Andrew T Hattersley, Timothy M Frayling, Andrew M Prentice

**Affiliations:** 1 MRC International Nutrition Group, NPHIRU, EPH, London School of Hygiene & Tropical Medicine, London WC1E 7HT, UK; 2 Medical Genetics Unit, Ospedale S. Pietro FBF, 00189 Rome, Italy; 3 MRC Keneba, Medical Research Council Laboratories, Fajara, PO Box 273, Banjul, Gambia; 4 Genetics of Complex Traits, Institute of Biomedical and Clinical Science, Peninsula Medical School, Exeter EX1 2LU, UK; 5 Diabetes Genetics, Institute of Biomedical and Clinical Science, Peninsula Medical School, Exeter EX1 2LU, UK

## Abstract

**Background:**

Variation in the fat mass and obesity associated (*FTO*) gene has been reproducibly associated with body mass index (BMI) and obesity in populations of White European origin. Data from Asians and African-Americans is less conclusive.

**Methods:**

We assessed the effect of 16 *FTO *polymorphisms on body mass in a large population of predominantly lean Gambians (N_max _2208) participating in a long-term surveillance program providing contemporary and early-life anthropometric measurements.

**Results:**

Sixteen *FTO *tagSNPs screened here, including several associated with BMI in Europeans, were not associated with birth weight (BWT), early weight gain in 1–2 year olds, BMI in adults (≥ 18 y), or weight-for-height (WFH) z-score across all ages. No association was seen between genotype and WFH z-score or other measures of body mass. The confidence limits indicate that the effect size for WFH z-score never exceeded 0.17 units per allele copy for any SNP (excluding the three SNPs with allele < 15%). with much the lowest allele frequency. The confidence interval of the effect size for rs9939609 did not overlap that reported previously in Europeans.

**Conclusion:**

To our knowledge this is the first study of *FTO *gene variation in a well-characterised African population. Our results suggest that *FTO *gene variation does not influence measures of body mass in Gambians living a traditional lifestyle, or has a smaller effect than that detected in Europeans. These findings are not directly comparable to results from previous studies in African-Americans due to differences in study design and analysis. It is also possible that any effect of *FTO *genotype on body mass is of limited relevance in a lean population where little excess food is available, compared to similar ethnic populations where food supply is plentiful.

## Background

Variation in the *FTO *(fat mass and obesity associated) gene has been shown to associate with body mass index (BMI) and predisposition to obesity in several European, Caucasian-American and Hispanic-American populations [1-7]. The observed effect size of about 0.35 kg/m^2 ^(0.1 z-score units for BMI) per susceptibility allele in all studies is substantial for a phenotype controlled by many genetic and environmental factors. These results have been replicated in several large sample sets of both children and adults. Most of these studies report on associations between BMI and one or more single nucleotide polymorphisms (SNP), with markers mapping to a stretch of about 50 kb within intron 1 of the *FTO *gene. None of these variants are known to have a direct functional effect so they are likely to be in linkage disequilibrium (LD) with the true causative variant [8]. Genome-wide association studies of type 2 diabetes have also suggested an involvement of *FTO *in the disease pathogenesis, although this is likely to reflect the association with the associated phenotype of obesity [1,9,10].

Data from studies in Asians did not initially seem to support a role of *FTO *variants in obesity in such populations [11,12]. However, several larger studies of Chinese, Malay, Korean and Japanese populations as well as Canadians of South Asian origin and Inuit demonstrated associations between *FTO *polymorphisms and BMI/obesity with similar effect sizes compared to populations of European ancestry [13-18]. The association between polymorphisms in the *FTO *gene and BMI could not be confirmed in an African-American population or an Oceanic population, but a more recent study in African-Americans reported some correlation [3,19,20].

We set out to assess variation in the *FTO *locus in a well-characterised Gambian study cohort and this is, to our knowledge, the first report on *FTO *in a native African population. Our study population is not comparable in terms of BMI range to most previously studied obese populations. However, we hypothesized that given the previously reported size of effect of FTO variation on BMI, effects within any range of BMI should be detectable even in our predominantly lean study population. We further hypothesized that an FTO-associated constitutive effect might be more apparent in a population exposed to a more homogeneous dietary environment. We studied 2208 Gambians from a rural subsistence farming population suffering seasonal energy stresses. We assessed associations with weight-for-height (WFH) z-score at different ages and with BMI in adults. Attention was also paid to see whether effects in early in life could be detected (weight gain in 1–2 year olds) or by sex. Since seasonal variations in maternal energy balance and fat mass have a profound influence on conception rates in this population [21,22] we further hypothesized that *FTO *might influence reproductive success and tested this using various indices of fertility.

## Methods

### Study participants

DNA samples were originally obtained for a study designed to investigate the association between genetic markers and birth and early life anthropometry (summary statistics shown in Table 1). Recruitment took place in 2002/3 and was based in three villages in West Kiang (Keneba, Kanton Kunda, and Manduar) in which an antenatal/maternity program had been running since 1974. All 2242 children whose mothers had participated in the Keneba antenatal scheme (i.e. for whom birth weight (BWT) had been recorded) and their parents, grandparents, siblings and other close relatives provided the source of potential subjects. This population is almost exclusively Mandinka and socially homogenous. The attrition of available data at each stage of the study are summarised in Additional file 1, Table S1. There were 7 pairs of monozygotic twins; one of each pair was randomly selected and dropped from the analysis to avoid bias. Records/census data on the inhabitants of the West-Kiang region since 1950 were available from the MRC Keneba database. This includes demographic and anthropometric data on ~10,000 individuals and was used for establishment of pedigree structures and determination of fertility indices. Ethics approval was granted by the joint Gambia Government/MRC Ethics Committee (SCC/EC L2007.46d) and all subjects and/or legal guardians provided written, informed consent.

**Table 1:** Summary statistics on weight, height and BMI by age and sex at time of DNA collection

	**N**		**mean (sd) weight-for-age** z-score*		**mean (sd) height-for-age** z-score*		**mean (sd) BMI**	
**age (y)**	**male**	**female**	**male**	**female**	**male**	**female**	**male**	**female**
1.75 – 5	105	91	-1.50 (0.93)	-1.34 (0.96)	-1.46 (1.18)	-1.23 (1.20)	15.34 (1.32)	15.01 (1.51)
5 – 10	194	155	-1.57 (0.97)	-1.54 (0.85)	-1.20 (0.90)	-1.20 (0.76)	14.41 (1.05)	14.28 (1.27)
10 – 15	152	171	-1.71 (1.00)	-1.46 (1.03)	-1.38 (1.03)	-1.15 (0.91)	15.72 (1.55)	16.48 (2.26)
15 – 20	150	195	-2.12 (1.05)	-0.80 (1.09)	-1.49 (1.17)	-0.80 (0.92)	17.80 (1.78)	20.51 (2.93)

			**mean (sd) weight, kg**		**mean (sd) height, cm**			

20 – 25	96	142	60.91 (6.63)	55.19 (7.79)	173.20 (7.51)	160.53 (5.73)	20.27 (1.72)	21.35 (2.70)
25 – 30	23	133	64.62 (10.11)	55.73 (11.28)	175.15 (6.74)	160.34 (6.76)	21.08 (2.90)	21.63 (3.97)
30 – 40	55	145	64.28 (10.51)	57.79 (10.42)	170.65 (7.27)	158.73 (5.37)	21.88 (2.75)	22.97 (4.02)
40 – 50	66	126	63.41 (11.08)	56.99 (13.07)	169.83 (5.45)	158.26 (6.83)	21.89 (3.31)	22.77 (4.42)
50 – 60	73	92	61.51 (10.12)	54.70 (10.21)	167.91 (6.18)	158.10 (5.50)	21.65 (3.15)	21.86 (3.48)
> 60	77	91	54.40 (8.23)	51.02 (9.40)	164.52 (6.34)	156.01 (6.14)	19.98 (2.61)	21.01 (3.05)
Total	991	1341						

* relative to UK standards [35]. UK reference data were used because the new WHO standards do not extend beyond 5 years of age.

### Genetic analysis

DNA was extracted from whole blood or PBMCs using a standard salting-out method according the protocol of the DNA Bank at the MRC Laboratories in The Gambia [23]. LD and haplotype blocks across the *FTO *locus were assessed in the publicly available HapMap datasets for Yoruba and Caucasians using the Haploview program (default parameters based on r-square; ; see Additional file 2, Figure S2) [24]. The SNP selection was confined to a region of high LD in Caucasians of 47 kb spanning exon 2 and parts of intron 1 and 2 either side [1]. We selected 19 tagging SNPs based on HapMap Yoruba data across this region using the 'Tagger' algorithm with a cut-off of r^2 ^≥ 0.8 and a minor allele frequency (MAF) of > 0.1. Three 'key' SNPs as identified through previous associations (rs9939609, rs17817449 and rs3751812) were force-included [1,2]. Genotyping, was performed by KBiosciences  using a modified Taqman (Applied Biosystems ) assay. Three SNPs failed at the design stage, and individuals with ≥ 50% missing genotype data were excluded (N = 177); the average genotyping failure rate across the remaining 16 markers was 6.47% (ranging from 5.23 to 8.04%) for our sample set of 2350 individuals. Divergence from Hardy-Weinberg Equilibrium (HWE) was assessed based on genotype data from the whole sample set. All SNPs were in HWE except for rs9935403 (P < 0.001), which is in line with the expected number diverging from HWE at the P = 0.05 level. Based on Yoruba HapMap data the 16 SNPs used for analysis tagged 60 of 87 alleles with a mean r^2 ^of 0.94 across the 47 kb region and 100% of all 87 alleles with r^2 ^> 0.8. For further details see Additional file 2, Figure S2.

### Statistical analysis

All statistical analysis was performed using Stata (version 9, ). The current study looked at the effect of genotype on two main outcomes: i) body mass at different ages and ii) indices of female fertility. A breakdown of sample numbers by outcome is shown in Additional file 1, Table S1. Each outcome was regressed separately on each SNP using, unless otherwise stated, a multi-level model to allow for intra-family correlation. The polygamous nature of this society and thus the complex pedigree structure, e.g. presence of a large proportion of half-sibs, made a standard family-based analysis extremely difficult. A tractable, if cruder, analysis was made possible by defining family groups as siblings sharing the same mother. Each outcome was regressed separately on each SNP using, unless otherwise stated, a multi-level model to allow for intra-family correlation. Where multi-level models were not practical, we made allowance for family clustering by estimating robust (Huber-White) standard errors [25]. For completeness we also analysed the data accounting for relatedness according to clustering by father. The inclusion of the random effect did not substantially change size of the estimated effect, reassuring us that any confounding due to family clustering was negligible. An additive genetic model was employed in all analyses (coding genotypes as 0, 1 and 2). A dominance term was added to each model to check the validity of this assumption but in no case was it found to be significant. We applied the Bonferroni correction to allow for multiple testing based on data for 16 SNPs, but not accounting for multiple outcomes. We are aware that this is too stringent regarding the number of markers and not stringent enough in terms of outcomes, however, our conclusions are unlikely to change employing a different correction factor. Each association was controlled for a number of covariates believed to influence the outcome: child's year of birth (as a linear trend) and village were always fitted and maternal height for both BWT and early weight gain, and sex for BWT and BMI. We also tested the sex-genotype interaction for each body mass outcome.

#### (i) Body mass

Body mass was assessed as BWT, early weight gain in 1–2 year olds, BMI in adults as well as a WFH z-score analysed over all ages.

##### Birth weight

BWT was fitted to genotype using generalised least squares regression [26]. Uniquely for this outcome we examined the effects of both offspring and maternal genotypes.

##### Early weight gain

We calculated an internal weight-for-age z-score (similar to the weight-for-height z-score described in Additional file 3, Methods S1) using all records from the routine child clinic in Keneba. For those individuals for whom weight measurements were recorded on at least five occasions during the first two years of life, with at least one in each of the first and second years of life, we regressed this weight-for-age z-score on age for each child and recorded the fitted slope and its standard error. Those children whose change in z-score could not be estimated accurately (standard error > 0.4) were excluded from the analysis. Finally, the association between the slopes of the remainder and genotype was examined using GLS regression.

##### Weight-for-height z-score

The study members covered a wide range of ages, including a large percentage (46%) less than 18 years. Below this age BMI is strongly age-dependent and unsuitable as a measure of body composition. Instead we constructed an internally calibrated WFH z-score (see Additional file 3, Methods S1). GLS regression was used to examine its association with genotype. Information on season of measurement was available and featured as covariates in this analysis.

##### BMI

BMI (wt/ht^2^, kg/m^2^) was analysed for adults (over 18 y) using GLS regression. Among adults this variable is closely related to the weight-for-height score of the previous section. It was included here to provide a direct comparison with previous work. Additional file 1, Table S3 gives a breakdown of BMI in our population.

#### (ii) Female fertility

We assessed age at first delivery, reproductive success and seasonality of birth. For further details regarding the methods applied please refer to the Additional file 3, Methods S1.

## Results

Summary statistics on body mass by age and sex are shown in Table 1. The results of regression analyses for WFH z-score for the whole sample are tabulated for each SNP in Table 2. Results on BWT, early weight gain and BMI for sample subsets are reported in Additional file 1, Table S2. We observed no association with WFH z-score or other measures of body mass despite reasonable precision. The standard error of the effect size (derived from multiple regression models with covariates) for the WFH z-score was approximately 0.033 units per allele copy, and the corresponding 95% confidence interval less than 0.17, for all SNPs. The confidence intervals for other measures of body mass and all but the three SNPs with the lowest allele frequencies (< 15%; rs3751812, rs9931494 and rs9935403) indicate that the *FTO *genotype is unlikely to be associated with differences per allele of greater than 0.12 in WFH z-score, 0.74 kg/m^2 ^in BMI among adults, 80 g in BWT and 0.11 z-scores difference in growth rate in the first 2 years of life. The exception was rs3751812, the SNP with much the lowest allele frequency (4.4%). The 95% confidence interval for the WFH z-score of rs9939609 (-0.061, 0.065) did not embrace the lower bound (0.08) for the equivalent effect on BMI z-score reported previously in Europeans [1].

**Table 2:** Results for (A) weight-for-height z-score and (B) reproductive success^§ ^for 16 SNPs in *FTO *gene

			**(A) weight-for-height zscore^1^**			**(B) Reproductive success^§2^**		
**SNP**	**base change**	**MAF [%]**	**coef**	**CI95%**	**p**	**% diff in birth rate**	**CI95%**	**p**
rs6499642	C/T	32.07	0.0373	(-0.0307, 0.1052)	0.2725	4.7%	(-1.0%, 10.9%)	0.1017
rs6499643	C/T	43.07	0.0561	(-0.0073, 0.1194)	0.0767	0.1%	(-5.0%, 5.5%)	0.9665
rs7206790	C/G	39.68	-0.0313	(-0.0950, 0.0324)	0.3262	-4.0%	(-9.0%, 1.4%)	0.1354
rs9940646	C/G	41.26	0.0145	(-0.0497, 0.0788)	0.6511	-0.7%	(-5.6%, 4.5%)	0.7842
rs12447107	C/G	31.31	0.0152	(-0.0526, 0.0831)	0.6534	-1.9%	(-7.0%, 3.5%)	0.4801
rs17817288	A/G	37.91	-0.0016	(-0.0678, 0.0646)	0.9612	4.3%	(-0.7%, 9.5%)	0.0845
rs16945088	A/G	40.91	0.0281	(-0.0365, 0.0927)	0.3846	5.1%	(0.2%, 10.1%)	0.0356
rs17817449	G/T	34.50	-0.0039	(-0.0700, 0.0621)	0.9052	-0.9%	(-5.6%, 4.0%)	0.7046
rs8063946	C/T	41.93	0.0127	(-0.0518, 0.0772)	0.6932	3.0%	(-1.7%, 7.9%)	0.2068
rs3751812	G/T	4.39	0.0164	(-0.1376, 0.1703)	0.8317	7.2%	(-2.7%, 18.1%)	0.1535
rs3751813	G/T	44.43	-0.0243	(-0.0883, 0.0397)	0.4471	4.2%	(-0.8%, 9.4%)	0.0929
rs9939609	A/T	35.54	0.0019	(-0.0614, 0.0653)	0.9512	-3.3%	(-7.7%, 1.3%)	0.1510
rs9931494	C/G	12.42	0.0479	(-0.0467, 0.1425)	0.3117	-1.5%	(-7.7%, 5.2%)	0.6475
rs7190492	A/G	23.07	-0.0397	(-0.1136, 0.0342)	0.2828	-1.9%	(-7.7%, 4.4%)	0.5421
rs7204609	C/T	46.76	-0.0168	(-0.0819, 0.0482)	0.6044	-3.1%	(-7.6%, 1.6%)	0.1787
rs9935403	G/A	15.00	0.0644	(-0.0194, 0.1482)	0.1244	-0.8%	(-7.2%, 6.0%)	0.8003

Key: SNP single nucleotide polymorphism; MAF minor allele frequency

^§^as number of babies born (interpreted taking account of the mother's age and duration of observation)

^1 ^Sample size ranged between 2127 and 2164 and the number of family groups between 954 and 966 depending on SNP. Family group sizes ranged between 1 and 8.

^2^ Sample size ranged between 657 and 672 and number of family groups between 484 and 495 depending on SNP. Family group sizes ranged between 1 and 5. Total exposure ranged between 420 and 428 "reproductive lifetimes" and the number of births between 2985 and 3062.

The tabulated p-values are not corrected for multiple testing. The Bonferroni correction to account for testing 16 SNPs and achieve significance at the 5% level requires that the uncorrected p-value should be < 0.0033. In no case is this cut-off reached.

We note that dropping covariates made little difference to the effect sizes measured. Reanalysis based on clustering by father (rather than mother) had no effect on our findings. The analysis accounting for possible sex differences did show that no genotype effect varied significantly between the sexes for any body mass outcome.

We found no association of *FTO *SNPs with reproductive success (as number of babies born, Table 1), nor with age at first delivery or seasonality of birth (see Additional file 1, Table S2). The confidence intervals indicate that we should have been able to detect a 10% difference in reproductive success.

## Discussion

We set out to test for an effect of *FTO *variation on measures of body mass in a population of predominantly lean Gambians from a rural subsistence farming environment. The availability of anthropometric measures spanning from birth to each subject's current age allowed us to investigate the possibility that putative associations between *FTO *and body mass may be differentially expressed during different ages. In this rural Gambian population fetal growth is generally constrained with additional seasonal stresses when mothers are in negative energy balance in the hungry season [22]. Infant growth is extremely poor as a consequence of nutrient deficits and high levels of infections, and children deteriorate sharply compared to international growth standards (see Table 1) [27]. Adults are generally very lean (see Additional file 1, Table S3) and suffer seasonal variations in energy balance amounting to up to 50% annual variation in adipose tissue mass [21]. We thus assessed separately BWT, early weight gain in 1–2 year olds, and BMI in adults (> 18 y), as well as a WFH z-score for the whole population. We used internally calibrated WFH z-score as outcome measure (see Additional file 3, Methods S1) because almost half of our study participants were under 18 years, and BMI is not suitable in young individuals. Our results on all four of these measures showed no association with SNPs in the first intron of the *FTO *gene in Gambians. The confidence intervals of our estimates indicate that there is a difference in effect size between Europeans and Africans, however, these populations are exposed to very different environments (e.g. nutrition) and any interaction between gene and environment could have given rise to differences in the size of genetic effect we measure. In the specific case of the association between variant rs9939609 and WFH z-score, the 95% confidence limits allow a maximum increase of 0.065 z-score units for each additional copy of the A allele. Previous studies have shown an increase in BMI of 0.10 z-score units per copy with a lower confidence bound of 0.08 [1], i.e. higher than our maximum. Similar effect sizes to those initially reported for FTO for the same or neighbouring polymorphisms have been reported by others in Europeans, Caucasian Americans and Hispanics, thus supporting a role for genetic variation in the *FTO *gene in affecting BMI across the range as well as obesity in such populations [2-7]. Most studies have focused on the comparison of obese versus non-obese individuals, and *FTO *is viewed as a 'fat' gene. However, the mode of action of FTO remains unknown and it is interesting to note that the A allele (favouring fatness) is the ancestral allele and the T allele has appeared more recently, encouraging the view that studies across different levels of fatness may help to uncover its mechanism of action.

*FTO *gene variants have also been assessed as determinants of fat mass around birth by three studies in Caucasians, neither of which found an effect on BWT [1,28,29]. The study by Lüpez-Bermejo also looked at early postnatal affects and reported an association of rs9939609 with cord blood visfatin, and weaker evidence for associations with weight and ponderal index, total, truncal and abdominal fat and weight gain in the first two weeks of life. The Danish longitudinal study, representing a wide range of BMI in adolescence (thus potentially overlapping more with our lean African population), reported the same SNP to be associated with increasing BMI z-score at all ages, weight gain from birth to age 7 and a second increase in BMI z-score from adolescence and early adulthood onwards [29]. Our analysis was not able to determine very early effects, although we did assess weight gain in 1–2 year olds, which did not appear to be affected by polymorphisms in the *FTO *gene. In a study of Swedish children Jacobsson *et al. *reported a differential effect of rs9939609 on BMI according to the sex of the child [30]. These authors did not, however, test this interaction statistically and indeed, given the large overlap between the confidence intervals of the ORs for boys and girls, it is unlikely that this difference was statistically significant. We saw no evidence for a genotype-sex interaction with any of the body mass measurements we recorded.

Initial studies in Asians showed no association between *FTO *variants and BMI in Han Chinese, an Oceanic population, Japanese or Chinese and Oji-Cree living in Canada [11,12,18,19]. However, other studies in Chinese, Koreans, Malay, Japanese, as well as Canadians of South Asian and Inuit origin, presented support for a role of polymorphisms in the *FTO *gene and obesity in such populations [13-18]. Although the allele frequency of rs9939609 is substantially lower in Asians, the effect size on BMI appears to be comparable to populations of European ancestry. It is thus possible that an effect could only be seen in the later larger studies which had more statistical power.

There appear only to be two studies in African-Americans to date and the findings from these are inconclusive. Scuteri *et al *reported a lack of association between *FTO *variation and BMI in 968 African-Americans and our data in Gambians are in line with these findings [3]. The study by Grant and colleagues described a correlation between a SNP (rs3751812, P = 0.017, OR = 1.3, 95%CI 1.1–1.6) in the *FTO *gene and obesity in young African-Americans (578 obese vs 1424 controls) [20]. However, none of the other 12 polymorphisms genotyped in this region were associated with obesity. The possibility that the result for rs3751812 may represent a false positive finding cannot be excluded at this stage. We did not replicate the observed association with rs3751812 in our Gambian population, however, a direct comparison is not possible due to the different nature of study populations and the use of a binary versus a quantitative outcome measure in Grant's and our study, respectively.

Discrepancy in observations from the above mentioned studies in African-Americans could indicate that these populations are admixed, i.e. genetically heterogeneous, as expected given their ancestry. This and differences between study populations (such as the study of obese/non-obese subjects), differences in the environment or population specific biases could also explain discrepancies in the comparison of data from different African-American studies or in the comparison with data from native Africans. We observed some differences in MAF for the rs3751812 variant, with similar frequencies around 10% African-Americans and around 4–6% in Gambians and Yoruba (YRI). (In our study rs3751812 had much the lowest allele frequency and hence our power to detect an association with BMI would have been weaker than for other SNP.) Yet, HapMap data comparing Yoruba and African-Americans in Grant's study show a broadly similar LD pattern across this locus (see Additional file 2, Figure S2 and figures in [20]). The LD heat map based on our own data in Gambians is also very similar to that in YRI.

The strength in our analytical approach is that our study population was recruited in the West Kiang region of the Gambia which is ethnically very homogenous (95% Mandinka), so population substructure is unlikely to affect our results. Furthermore, given the availability of the large amount of anthropometric data for the majority of our 2208 study participants measured at numerous time points we were able to assess a possible role of *FTO *variation on body mass at different ages.

Our second prior hypothesis was that genetic variation in the *FTO *gene might affect fertility given the strong influence of body fat on female reproductive function that is readily detectable within this population [31]. Given that we did not detect a correlation with measures of body mass in our study, it is maybe not surprising that we observed no association with fertility-related phenotypes and *FTO *SNPs in our analysis.

## Conclusion

Despite having sufficient power to detect a difference of only 0.065 z-score for BMI our study failed to detect associations between *FTO *polymorphisms and a range of measures of body mass across the life-course (BWT, early weight gain, BMI and WFH z-score) or fertility in Gambians. There can be two explanations for such a result: either that there truly is no/little association, or that an underlying constitutive effect is only revealed by an obesogenic environment. Suggestions that FTO may operate by modulating appetite [32-34] would potentially support the latter view, and may be clarified by further studies in overweight and obese native Africans. To the best of our knowledge this is the first report on genetic variation in the *FTO *gene in a native African population. Given that highest levels of genetic diversity (i.e. low LD) exists in Africa, such studies might also assist in fine-mapping causative variants and mechanisms.

## Abbreviations

BMI: body mass index [35]; BWT: birth weight; FTO: fat mass and obesity associated; HWE: Hardy-Weinberg equilibrium; LD: linkage disequilibrium; MAF: minor allele frequency; OFRE: observed fraction of reproductive effort; SNP: single nucleotide polymorphism; WFH: weight-for-height; YRI: Yoruba.

## Competing interests

The authors declare that they have no competing interests.

## Authors' contributions

AJF extracted previous data for this sample cohort, carried out all statistical analyses and drafted the manuscript. BJH coordinated and designed the genetic aspects of the study and drafted the manuscript. GS coordinated the DNA sample collection and helped draft the manuscript. PRS carried out the field work including sample and data collection. ATH helped draft the manuscript. TMF participated in the design of the study, coordinated the genotyping and helped draft the manuscript. AMP conceived of the study and drafted the manuscript. All authors read and approved the final manuscript.

## Pre-publication history

The pre-publication history for this paper can be accessed here:



## Supplementary Material

Additional file 1 **Tables S1–S3.** Table S1 gives a sample size breakdown by outcome measure and reason for inclusion; in Table S2 we present additional results for 16 *FTO *SNPs on (A) body mass as BWT (mothers' and babies' genotypes), early weight gain and BMI and (B) indices of fertility as age at first delivery and seasonality and Table S3 shows the distribution of BMI in Gambian study population. Click here for file

Additional file 2 **Figure S2: Location of polymorphisms genotyped across *FTO *locus and Linkage disequilibrium structure in different populations.** Heat maps based on Gambian and HapMap YRI comparative data of the LD structure of a section of the *FTO *gene is shown in Figure S2. Click here for file

Additional file 3 **Methods S1: Detailed statistical methods of measures of body mass and reproductive success.** The data provided represents the construction of weight-for-height z-score, analysis of female fertility (including age at first delivery, and seasonality of birth). Figure S1 shows the empirical distribution functions for births before and after 1980 and the fitted distribution function based on the pooled data. Click here for file
