# Supplementary material for: FTO gene variation and measures of body mass in an African population
Source: BMC Med Genet. 2009 Mar 5;10:21. doi: 10.1186/1471-2350-10-21 (PMC2666669; doi:10.1186/1471-2350-10-21)
Supplement: Additional file 1 — Tables S1–S3. Table S1 gives a sample size breakdown by outcome measure and reason for inclusion; in Table S2 we present additional results for 16 FTO SNPs on (A) body mass as BWT (mothers' and babies' genotypes), early weight gain and BMI and (B) indices of fertility as age at first delivery and seasonality and Table S3 shows the distribution of BMI in Gambian study population. [file 1471-2350-10-21-S1.pdf]

Additional file 1

Methods S1: Detailed statistical methods of measures of body mass and reproductive success.

Construction of weight-for-height z-score.

The data were divided into four subsets by age (above or below 18 years) and sex. Within each group the logarithm of weight was regressed on the first four orthogonal polynomials in both age and height and the first two pairs of Fourier terms to model season of observation. The interaction between the first order terms in age and height was also fitted since there were subtle changes in the weight-height relationships with age. The squared residuals from these analyses were then also regressed on orthogonal polynomials in age (only of degree two this time), using a generalised linear model with a gamma error structure, in order to estimate age-specific variance. The z-score for each individual was then calculated by dividing the residual of the log(wt) regression by the square root of the predictor from the gamma regression. No allowance for skewness was judged necessary since the use of the logarithm of weight yielded a residual distribution that was already symmetrical. The polynomials in age and height were adjudged to provide a more than adequate fit – no higher degree term were significant and no trend in the residuals was detected visual when plotted against age or height. We noted that among adults (over 18y), BMI and our weight-for-height z-score correlated strongly (R-sq=0.81) although not perfectly, largely because the average BMI is much greater in women than men in this population and this difference is accommodated by the z-score. The relationship is approximately linear: BMI=21.4+3.1*z-score.

Analysis of female fertility.

We assessed age at first delivery, reproductive success and seasonality of birth.

Age at first delivery: Only women who had been observed since before the age of 12 years were included in this analysis. Cox proportional hazard regression, with analysis scale being the woman's age, was used to allow for censoring: women were regarded as exposed up until the time they either gave birth to their first baby or were no longer under observation or the woman reached the age of 25 years. (Censoring at 25y was introduced in order the focus principally on the age at which women became fecund.) Robust standard errors were calculated.

Reproductive success: This was calculated as number of live babies delivered and interpreted by taking account of the mother's age and duration of observation. Briefly, women (>18 years of age in 2003) with a recorded first and last date of being "under observation" in Keneba were included in the analysis. Each woman's "observed fraction of reproductive effort" (OFRE) was calculated as the weighted proportion of her reproductive lifetime observed. A Poisson model fitting the number of babies born to each woman during her interval of observation was applied to assess the association between fertility and genotype. We applied robust standard errors (Huber-White sandwich estimator) to adjust for unmodelled influences on fertility. The Poisson model was fitted by the GEE method in order to allow for the correlation between members of the same family [1].

While the overall fertility in Keneba has declined, the distribution of the ages at which women deliver their babies has not changed noticeably since the 1950s when observations first began. The distribution of mothers age for births before and after 1980 are essentially identical - see supplementary Figure 1. The distribution function for maternal age at birth, F(age), was estimated using all 7133 recorded births to women of known age in Keneba between 1950 and 2006. The empirical distribution function, F(age), was determined at a particular age as the

proportion of all babies who were born to mothers of that age or younger. Thus F[x]=Pr(mother's age at delivery≤x). An algebraic formula closely approximating this function was derived by fitting logit[F(age)] to fractional polynomials (degree 3) in age. The difference between the values of this function at the ages at which a woman was first and last under observation gives an estimate of fraction of her lifetime's reproductive output we might on average expect to observe during her period of observation. We refer to this fraction as the "observed fraction of reproductive effort" (OFRE). Unfortunately frailty models (i.e. multi-level survival models) failed to converge for these data so robust standard errors were calculated instead.

For each female study member who had been genotyped and was over the age of 18 years in 2003, we recorded the date at which she was first and last "under observation" in Keneba, i.e. those dates between which we would have recorded any babies that she delivered. From these dates we calculated her "observed fraction of reproductive effort" (OFRE): the proportion of her reproductive lifetime that we observed, weighted to take account of the average fertility of women of her age over that interval (see Supplementary Methods 2 and Supplementary Figure 1). We excluded any woman whose interval of observation was unknown (N=62) or for whom we observed less than 10% of her "reproductive effort" (N=169). (This latter criterion was necessary to avoid failure of the likelihood approximations. The analysis was insensitive to the exact value of the cut-off). The association between fertility and genotype was investigated by fitting the number of babies born to each woman during her interval of observation with a Poisson model. Allowance was made for the duration and age that the woman was under observation by specifying her exposure to be her OFRE (i.e. by offsetting the linear predictor by log(OFRE)). Although the data followed a Poisson distribution quite closely, it is not justified to assume that it will necessarily be an exact model for the numbers of births. The spacing of pregnancies, for instance, is likely to be more uniform than random

if only because a woman cannot become pregnant again until she delivers her first child. This would tend to make the counts under-dispersed relative to the Poisson; unmodelled influences on fertility might have the opposite effect on dispersion. The net result was slight under-dispersion. We therefore applied robust standard errors (Huber-White sandwich estimator). Finally, the Poisson model was fitted by the GEE method in order to allow for the correlation between members of the same family [1].

Seasonality of birth. There is a strong seasonal fluctuation in birth frequency in this area of The Gambia which appears to be driven by fluctuations in maternal energy supply [2, 3]. The fewest births occur from May to August; nine months following maternal weight loss during the annual hungry/farming season. We used binomial regression to test seasonality of birth, i.e. whether the probability that a woman would deliver during these months (as surrogate for capacity to conceive under conditions of negative energy balance between August and November) was associated with her genotype. Our data was adequate to estimate the seasonal pattern – the seasonal terms were comfortably significant. Robust standard errors were fitted.

1. Liang K-Y, Zeger SL: **Longitudinal data analysis using generalized linear models**. *Biometrika* 1986, **73**:13-22.
2. Prentice AM: **Starvation in humans: evolutionary background and contemporary implications**. *Mech Ageing Dev* 2005, **126**(9):976-981.
3. Rayco-Solon P, Fulford AJ, Prentice AM: **Differential effects of seasonality on preterm birth and intrauterine growth restriction in rural Africans**. *Am J Clin Nutr* 2005, **81**(1):134-139.

Figure S1: Distribution function for mothers' age at delivery

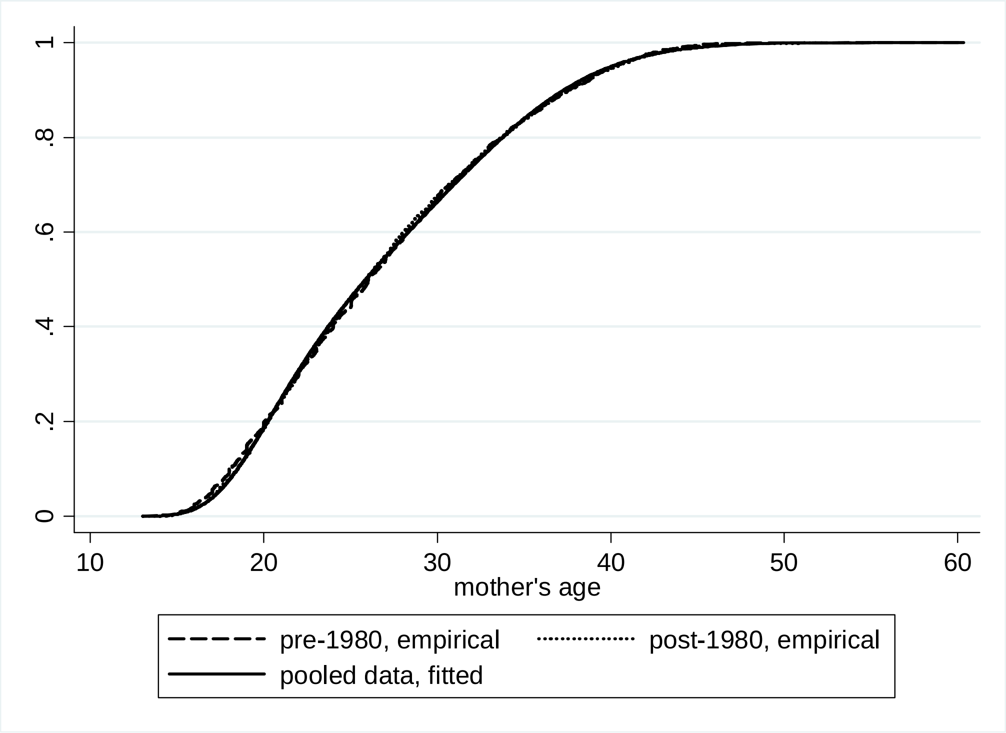

Figure S1 plots the empirical distribution functions for births before and after 1980 and the fitted distribution function based on the pooled data (based on all births in the three villages between 1950 and 2006). No difference in the distribution was observed pre- and post-1980, the mid point of available data.
