# Supplementary material for: FTO gene variation and measures of body mass in an African population
Source: BMC Med Genet. 2009 Mar 5;10:21. doi: 10.1186/1471-2350-10-21 (PMC2666669; doi:10.1186/1471-2350-10-21)
Supplement: Additional file 2 — Figure S2: Location of polymorphisms genotyped across FTO locus and Linkage disequilibrium structure in different populations. Heat maps based on Gambian and HapMap YRI comparative data of the LD structure of a section of the FTO gene is shown in Figure S2. [file 1471-2350-10-21-S2.pdf]

Additional file 2

Figure S2: **Location of polymorphisms genotyped across *FTO* locus and Linkage disequilibrium structure in different populations**

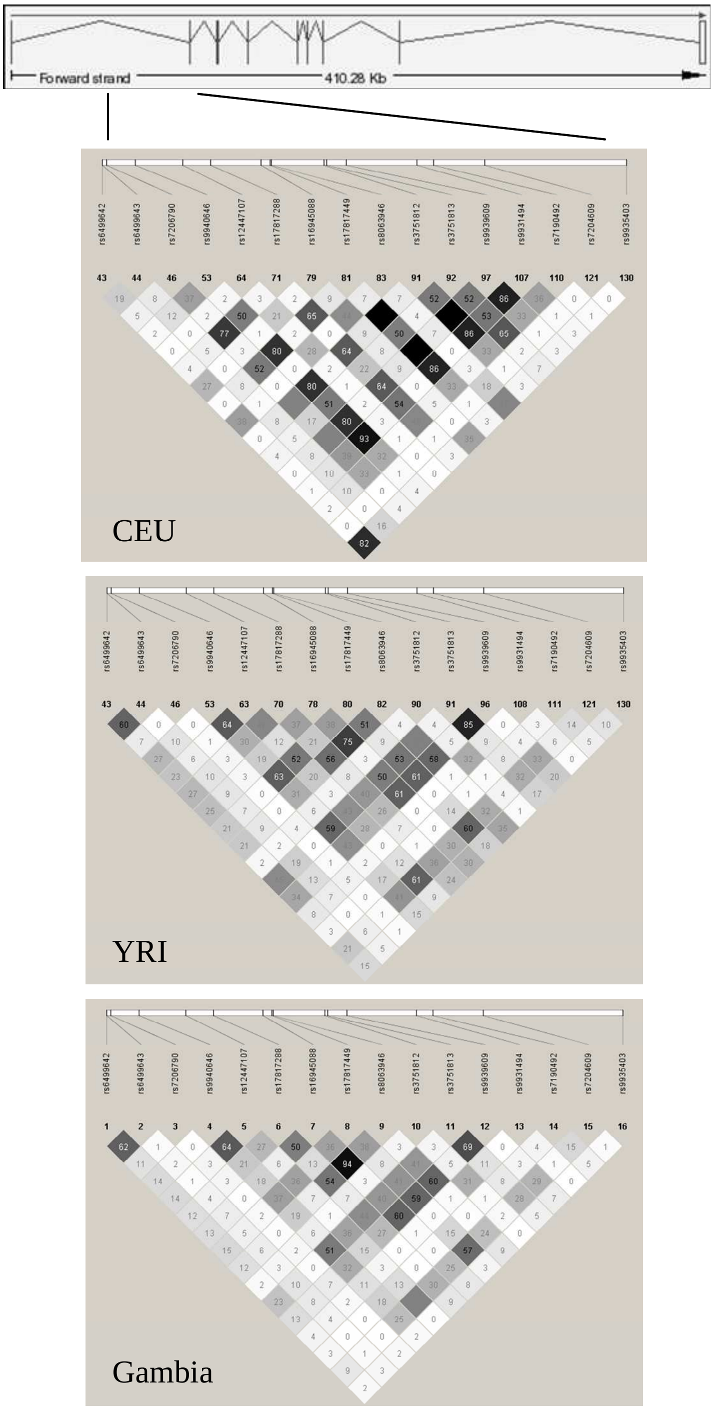

Key: CEU Centre d'Etude du Polymorphisme Humain; YRI Yoruba of Nigeria

Figure S2 shows heat maps of a section of the *FTO* gene with the location of our genotyped markers, and LD (as r^2 ranging from 0 to 1) derived from HapMap genotype data (release 21, July 2006) from 30 US family trios of Northern and Western European origin collected by the Centre d'Etude du Polymorphisme Humain (CEU) and 30 Yoruba family trios from Ibadan, Nigeria (YRI) [1]. LD plot for Gambian data based on 2350 individuals genotyped for 16 SNPs across the *FTO* gene.

1. Consortium IH: **A haplotype map of the human genome**. *Nature* 2005(437):1299-1320.
