# Supplementary material for: FTO gene variation and measures of body mass in an African population
Source: BMC Med Genet. 2009 Mar 5;10:21. doi: 10.1186/1471-2350-10-21 (PMC2666669; doi:10.1186/1471-2350-10-21)
Supplement: Additional file 3 — Methods S1: Detailed statistical methods of measures of body mass and reproductive success. The data provided represents the construction of weight-for-height z-score, analysis of female fertility (including age at first delivery, and seasonality of birth). Figure S1 shows the empirical distribution functions for births before and after 1980 and the fitted distribution function based on the pooled data. [file 1471-2350-10-21-S3.pdf]

Additional file 3

Table S1: Sample size breakdown by outcome measure and reason for inclusion

	Principle relationship to index[§] child *					
	**index**	**parent**	**sibling**	**grandparent**	**other**	**total**
Individual with known birth weight	2242	-	-	-	-	2242
Bled for DNA and successfully genotyped for *FTO*	1379	731	69	120	51	2350
**i) Samples available body mass analyses:**						
Birth weight	1379	0	0	0	0	**1379**
Early weight gain - sufficient post-natal weight measurement to estimate this accurately	1,194	68	48	0	7	**1317**
Weight-for-height analysis - weight and height recorded	1294	685	56	95	45	2175
BMI - weight and height recorded and age >18 years	312	684	23	95	35	1149
**ii) Samples available fertility analyses:**						
Age at first delivery - monitored by the midwives since the age of 14yr	79	325	1	18	6	**429**
Reproductive success as number of babies born - females >18 years monitor by midwives	164	422	5	75	12	**673**
Season of birth - women who have delivered at least one baby of known date of birth	154	477	8	87	15	**741**

[§] Index children are those children born in the three core villages and for whom birth weight and precise date of birth were recorded.

Table S2: Additional results for 16 *FTO* SNPs on (A) body mass as BWT (mothers' and babies' genotypes), early weight gain and BMI and (B) indices of fertility as age at first delivery and seasonality

**(A)**	**BWT (infant's genotype) [1]**			**BWT (mother's genotype) [2]**			**BMI (≥18yr only) [3]**			**Early weigh gain [4]**		
**SNP**	**coef**	**CI95%**	**p**	**coef**	**CI95%**	**p**	**coef**	**CI95%**	**p**	**coef**	**CI95%**	**p**
rs6499642	0.0141	(-0.0241, 0.0523)	0.4602	-0.0140	(-0.0652, 0.0372)	0.5847	0.1029	(-0.2115, 0.4173)	0.5128	-0.0157	(-0.0685, 0.0372)	0.5529
rs6499643	0.0262	(-0.0096, 0.0620)	0.1436	0.0093	(-0.0391, 0.0577)	0.7006	0.1778	(-0.1172, 0.4728)	0.2281	-0.0401	(-0.0897, 0.0096)	0.1063
rs7206790	0.0032	(-0.0323, 0.0387)	0.8563	0.0181	(-0.0300, 0.0662)	0.4519	-0.0926	(-0.3951, 0.2100)	0.5405	0.0094	(-0.0402, 0.0590)	0.7050
rs9940646	0.0093	(-0.0258, 0.0445)	0.5964	-0.0275	(-0.0772, 0.0222)	0.2681	-0.0349	(-0.3430, 0.2732)	0.8210	-0.0022	(-0.0505, 0.0461)	0.9267
rs12447107	0.0143	(-0.0233, 0.0519)	0.4479	0.0063	(-0.0453, 0.0579)	0.8085	-0.0798	(-0.4042, 0.2446)	0.6226	0.0265	(-0.0257, 0.0786)	0.3102
rs17817288	0.0093	(-0.0286, 0.0472)	0.6239	-0.0001	(-0.0466, 0.0464)	0.9968	0.0241	(-0.2839, 0.3321)	0.8756	-0.0174	(-0.0702, 0.0354)	0.5102
rs16945088	0.0186	(-0.0176, 0.0547)	0.3040	-0.0007	(-0.0489, 0.0474)	0.9752	0.1159	(-0.1887, 0.4205)	0.4466	-0.0162	(-0.0667, 0.0343)	0.5204
rs17817449	-0.0055	(-0.0424, 0.0315)	0.7671	-0.0178	(-0.0660, 0.0304)	0.4601	-0.1799	(-0.4867, 0.1270)	0.2410	0.0307	(-0.0208, 0.0823)	0.2328
rs8063946	0.0220	(-0.0141, 0.0581)	0.2225	0.0070	(-0.0400, 0.0540)	0.7664	0.0514	(-0.2495, 0.3523)	0.7328	-0.0014	(-0.0522, 0.0493)	0.9555
rs3751812	-0.0202	(-0.1038, 0.0634)	0.6288	-0.1210	(-0.2251, -0.0168)	0.0202	-0.3999	(-1.1125, 0.3128)	0.2618	-0.1279	(-0.2414, -0.0145)	0.0241
rs3751813	-0.0031	(-0.0379, 0.0318)	0.8609	0.0403	(-0.0080, 0.0886)	0.0952	0.0556	(-0.2523, 0.3635)	0.7181	-0.0122	(-0.0609, 0.0365)	0.6165
rs9939609	0.0045	(-0.0303, 0.0392)	0.7971	-0.0213	(-0.0696, 0.0270)	0.3774	-0.0631	(-0.3626, 0.2364)	0.6735	0.0095	(-0.0390, 0.0580)	0.6961
rs9931494	0.0067	(-0.0457, 0.0590)	0.7993	**-0.0843**	**(-0.1469, -0.0217)**	**0.0071**	-0.1592	(-0.6004, 0.2819)	0.4703	-0.0379	(-0.1110, 0.0351)	0.2990
rs7190492	-0.0074	(-0.0487, 0.0339)	0.7209	-0.0203	(-0.0790, 0.0383)	0.4884	-0.1737	(-0.5253, 0.1779)	0.3232	-0.0578	(-0.1149, -0.0006)	0.0432
rs7204609	-0.0014	(-0.0380, 0.0353)	0.9408	0.0308	(-0.0175, 0.0792)	0.2021	-0.1369	(-0.4460, 0.1723)	0.3759	0.0598	(0.0088, 0.1108)	0.0191
rs9935403	-0.0015	(-0.0491, 0.0462)	0.9514	0.0433	(-0.0180, 0.1047)	0.1579	0.3654	(-0.0129, 0.7438)	0.0534	0.0049	(-0.0619, 0.0716)	0.8843

[1] Sample size ranged between 1133 and 1151 and the number of family groups between 456 and 462 depending on SNP. Family group sizes ranged between 1 and 8.

[2] Sample size ranged between 975 and 1011 and number of family groups between 667 and 689 depending on SNP. Family group sizes ranged between 1 and 8.

[3] Sample size ranged between 1119 and 1167 and the number of family groups between 667 and 689 depending on SNP. Family group sizes ranged between 1 and 7.

[4] Sample size ranged between 1126 and 1145 and the number of family groups between 427 and 432 depending on SNP. Family group sizes ranged between 1 and 8.

(B)	Age at first delivery* [5]			Seasonality of birth [6]		
SNP	coef	CI95%	p	OR	CI95%	p
rs6499642	-10.33%	(-22.77%, 4.12%)	0.1446	1.0075	(0.8892, 1.1416)	0.9043
rs6499643	-12.03%	(-23.38%, 0.99%)	0.0633	1.0443	(0.9252, 1.1788)	0.4741
rs7206790	-2.10%	(-16.55%, 14.85%)	0.7907	1.0853	(0.9681, 1.2167)	0.1519
rs9940646	-3.82%	(-16.89%, 11.30%)	0.5935	1.0874	(0.9612, 1.2303)	0.1745
rs12447107	2.76%	(-11.18%, 18.89%)	0.7087	1.0716	(0.9438, 1.2168)	0.2759
rs17817288	4.90%	(-10.29%, 22.68%)	0.5407	1.0161	(0.9004, 1.1466)	0.7920
rs16945088	-1.11%	(-15.10%, 15.19%)	0.8839	1.0557	(0.9432, 1.1816)	0.3359
rs17817449	6.16%	(-9.13%, 24.01%)	0.4421	1.0763	(0.9550, 1.2130)	0.2185
rs8063946	-0.36%	(-14.18%, 15.68%)	0.9615	1.0227	(0.9148, 1.1435)	0.6868
rs3751812	4.60%	(-26.03%, 47.92%)	0.7951	1.0202	(0.8088, 1.2870)	0.8631
rs3751813	-0.77%	(-14.38%, 15.00%)	0.9160	0.9885	(0.8736, 1.1184)	0.8508
rs9939609	7.38%	(-7.25%, 24.31%)	0.3311	1.0467	(0.9271, 1.1817)	0.4522
rs9931494	4.12%	(-17.76%, 31.82%)	0.7321	0.9579	(0.8126, 1.1293)	0.6013
rs7190492	2.59%	(-13.37%, 21.50%)	0.7621	0.9613	(0.8336, 1.1086)	0.5798
rs7204609	0.50%	(-12.65%, 15.63%)	0.9435	1.0091	(0.8973, 1.1348)	0.8778
rs6499642	-10.33%	(-22.77%, 4.12%)	0.1446	1.0075	(0.8892, 1.1416)	0.9043
rs6499643	-12.03%	(-23.38%, 0.99%)	0.0633	1.0443	(0.9252, 1.1788)	0.4741
rs9935403	2.95%	(-15.57%, 25.54%)	0.7692	0.9247	(0.7897, 1.0828)	0.3214

[5] Sample size ranged between 417 and 428.

[6] Sample size ranged between 721 and 738.

* Fitted to a proportional hazard model: the coefficient indicates the proportional difference (per allele copy) in the “instantaneous hazard” of a woman delivery her first child at a given age.

The tabulated p-values are not corrected for multiple testing. While it is inevitable that some SNPs were significantly associated (p<0.05) with some of the six outcomes assessed in this study, the number of such associations was no greater than might be expected given the number of tests applied. Furthermore, no one association achieved a p-value less than 0.0033 as required by the Bonferroni correction for the best of 16 tests and significance at the 5% level.

Table S3: Distribution of BMI in Gambian study population

Sex	Age group [y]	BMI <18	18-25	25-30	>30	N (all)
F	0 - 10	97.80	1.50	0.00	0.70	267
	10 - 18	61.70	35.70	2.30	0.30	308
	18 - 30	8.70	79.40	9.30	2.50	355
	30 - 45	7.70	69.40	17.10	5.90	222
	45 - 60	9.80	69.90	14.40	5.90	153
	> 60	19.40	68.40	10.20	2.00	98
M	0 - 10	98.10	1.60	0.00	0.30	321
	10 - 18	83.80	15.00	0.00	1.20	253
	18 - 30	14.90	83.30	1.80	0.00	168
	30 - 45	7.40	81.90	8.50	2.10	94
	45 - 60	4.80	81.70	11.50	1.90	104
	>60	20.20	70.20	6.00	3.60	84
**all over 18 yr**						
**F**	> 18	9.90	73.70	12.40	4.00	828
**M**	> 18	12.00	80.20	6.20	1.60	450
